# Evaluation criteria for diagnosing motoric cognitive risk syndrome: a scoping review

**DOI:** 10.1590/1980-5764-DN-2024-0208

**Published:** 2025-05-19

**Authors:** Luisa Veríssimo Sampaio, Gislane Ferreira de Melo, Yandra Lima, Angela Maria Sacramento, Hudson Azevedo Pinheiro, Gustavo de Azevedo Carvalho

**Affiliations:** 1 Universidade Católica de Brasília, Brasília DF, Brazil.; 2 Centro Universitário LS, Brasília DF, Brazil.; 3 Secretaria de Estado de Saúde do Distrito Federal, Brasília DF, Brazil.

**Keywords:** Mental Status and Dementia Tests, Cognition, Gait, Diagnosis, Public Health, Testes de Estado Mental e Demência, Cognição, Marcha, Diagnóstico, Saúde Pública

## Abstract

Dementia encompasses a range of cognitive disorders marked by declining abilities in attention, memory, and social cognition. Considering this theme, motoric cognitive risk syndrome arises as a promising response to long-term care and early dementia detection although it is a pre-dementia stage.

**Objective:**

Considering that the early identification of individuals at higher risk of developing dementia could be key for the development of preventive actions, we conducted a scoping review to investigate the instruments used for the diagnosis of motoric cognitive risk syndrome.

**Methods:**

we searched ten electronic databases for studies presenting the assessment of motoric cognitive risk syndrome published between 2019 and 2023. Two independent reviewers screened studies according to including criteria using Mendeley Desktop software to collect, eliminate duplicates and facilitate full-text readings.

**Results:**

From an initial 225 publications related to motoric cognitive risk syndrome, 67 studies were considered eligible for full-text review. Some of those studies presented information for more than one sample, totalizing 82 studies in this scoping review.

**Conclusion:**

Although the diagnostic criteria to identify motoric cognitive risk syndrome are well defined in the literature, there is no description of a structured protocol or list of instruments recommended to evaluate this condition.

## RESUMO.

A demência abrange uma série de distúrbios cognitivos marcados pelo declínio das habilidades de atenção, memória e cognição social. Considerando-se este tema, a síndrome de risco cognitivo motor surge como resposta promissora aos cuidados de longa duração e à detecção precoce da demência, embora seja uma fase pré-demência.

**Objetivo:**

Considerando-se que a identificação precoce de indivíduos com maior risco de desenvolver demência pode ser crucial para o desenvolvimento de ações preventivas, realizamos uma revisão de escopo para investigar os instrumentos utilizados para o diagnóstico da síndrome de risco cognitivo motor.

**Métodos:**

Pesquisaram-se dez bases de dados eletrônicas em busca de estudos que apresentassem a avaliação da síndrome de risco cognitivo motor publicados entre 2019 e 2023. Dois revisores independentes selecionaram os estudos de acordo com critérios de inclusão usando o *software* Mendeley Desktop para coletar, eliminar duplicatas e facilitar a leitura do texto completo.

**Resultados:**

Do total inicial de 225 publicações relacionadas à síndrome de risco cognitivo motor, 67 estudos foram considerados elegíveis para a revisão de texto completo. Alguns desses estudos apresentaram informações para mais de uma amostra, totalizando 82 estudos nesta revisão de escopo.

**Conclusão:**

Embora os critérios diagnósticos para a identificação da síndrome de risco cognitivo motor estejam bem definidos na literatura, não há descrição de protocolo estruturado ou lista de instrumentos recomendados para avaliar essa condição.

## INTRODUCTION

Global aging presents numerous challenges, with dementia emerging as a significant concern that affects the elderly, their families, and communities. Dementia encompasses a range of cognitive disorders marked by declining abilities in attention, memory, and social cognition. It requires ongoing care due to its progressive nature, often leading to loss of autonomy^1-3^.

Likewise, the motoric cognitive risk syndrome (MCR) arises as a promising response to long-term care and early dementia detection. Characterized as a pre-dementia stage, MCR is detected by self-reported cognitive impairments concomitant with slow gait speed (GS) in older adults free from dementia diagnosis^4-6^. Notably, individuals identified with this syndrome exhibit a substantial propensity for progressing to dementia, especially vascular dementia^7,8^.

On the other hand, there is still no consensus on which assessment tools are most sensitive for measuring each category (subjective cognitive complaint and GS), which contributes to the heterogeneity in studies. Moreover, many studies only analyze the frequency of individuals with decreased GS and cognitive complaints in consortium samples, which limits the development of an effective methodology for selecting assessment tools^9^.

Incorporating the MCR evaluation into the public health system could not only help recognize individuals with the potential to develop dementia but also identify elderly individuals vulnerable to falling, disability, and hospitalization, which are significant challenges in elderly care^10-12^.

Based on this premise, we encounter the following guiding question: What assessment instruments are utilized to identify motor risk syndrome? Considering that, the purpose of this scoping review was to investigate the instruments used for the diagnosis of MCR in studies published between 2019 and 2023. Additionally, this review also intended to assess the presence of a protocol or a recommended list of instruments for this evaluation. Furthermore, the aim of this review was to identify the assessment tools used for evaluating subjective cognitive complaints and GS, applied in the identification of MCR.

## METHODS

### Study selection

The inclusion criteria were as follows: the population consisted of community-dwelling older adults aged 60 years or older, exposure was MCR assessment, and concept was diagnostic criteria and investigation approach. Studies that were not available in full were excluded, as were studies without a detailed description of the assessment instruments, review articles, and works exploring other related subjects such as cognitive decline, dementia syndromes, or GS.

### Data extraction and statistical analysis

To conduct this scoping review, the procedures recommended by the Joanna Briggs Institute (JBI) Systematic Review Network^13^ were employed, in addition to adhering to the guidelines outlined in the Preferred Reporting Items for Systematic Reviews and Meta-Analyses (PRISMA)^14^. This study was registered on the Open Science Framework (OSF) available on the identifier DOI 10.17605/OSF.IO/DGZR8.

For the identification of eligible journals in this research, the following databases were utilized: United States National Library of Medicine (PubMed), EBSCO's discovery service, *Biblioteca Virtual em Saúde* (BVS), Scientific Electronic Library Online (SciELO), Cochrane, Physiotherapy Evidence Database (PEDro), Wiley, ScienceDirect, Elsevier, and Web of Science. As a search strategy, "motoric cognitive risk syndrome" was defined as the keyword and the descriptors contained in the Medical Subject Headings (MeSH) and Descriptors in Health Sciences (DeCS) were "gait speed"/"gait speed"; "cognitive dysfunction"/"cognitive dysfunction" and "dementia"/"dementia".

Two reviewers collaborated to organize a data extraction chart aimed at gathering pertinent details from the studies included in the analysis. In the first phase of data extraction, the reviewers created a shared spreadsheet using Microsoft Excel to record the following information:

- database;
- citation and year;
- link to access the journal.

Subsequently, in the second phase, searches for available literature on MCR based on the provided information were conducted. During this phase, reviewers identified the title and subsequently conducted abstract readings to select studies. In the second phase, the pre-selected articles underwent full-text readings to assess adherence to inclusion criteria. At the conclusion of this process, researchers compared the selected studies and determined consensus. For the third phase of the research, eligible articles were stored in Mendeley Desktop software to eliminate duplicates and facilitate full-text readings.

**Figure 1:**
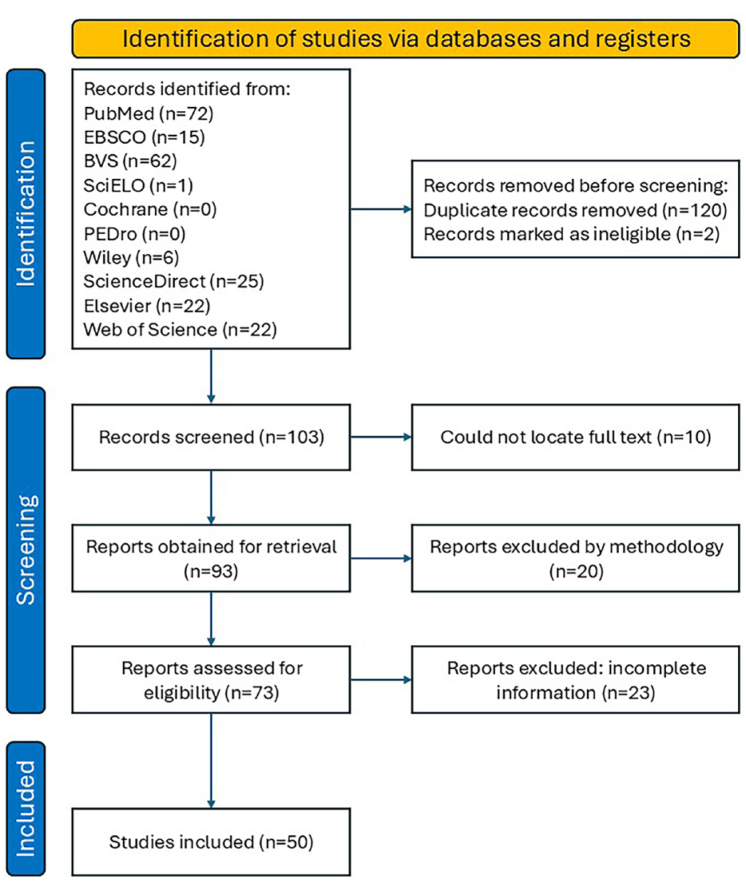
Prisma flowchart diagram.

Finally, two tables were developed, with the first focused on article identification, research objectives, and sample characteristics. The second table presents information related to the assessment instruments for detecting MCR, considering the subjective cognitive complaints, GS, functional capacity, and the absence of dementia. Following the selection, a descriptive analysis was conducted to establish, summarize, and elucidate the results pertinent to the objectives of this scoping review. The article selection process adhered to the PRISMA 2020 Flowchart guidelines (Figure 1).

## RESULTS

Firstly, a preliminary search for papers published between 2019 and 2023 was conducted, resulting in the identification of 225 articles related to MCR. After a full-text review, 50 studies were included in the scoping review. From those selected articles, nine^15-23^ presented results derived from multiple samples from different cohort studies conducted in various locations. For a more in-depth investigation of the information, these studies were accounted for separately, resulting in a total of 72 articles. The studies reviewed were described in Supplementary Material 1 (available at https://www.demneuropsy.com.br/wp-content/uploads/2025/01/DN-2024.0208-Supplementary-Material-1.docx) Table S1 ^6,9,12,15-61^.

### Motoric cognitive risk syndrome assessment tools

The assessment tools used to identify subjective cognitive complaints in the identification of MCR are in Supplementary Material 1 Table S2.

Regarding the assessment of subjective cognitive complaints for identifying individuals with MCI, no standardization was identified across the studies. It was observed that, for identifying subjective cognitive complaints, the authors used the following assessment instruments: one self-reported question^6,9,15,18,22-24,26,28,39,43-49,53,54,57,59-61^, a combination of self-reported question and cognitive testing^16,17,19, 20,22,30,32,34-36,55,56^, two or more self-reported questions^21-23,31,33,37,38,40,50,51,58^, and cognitive testing alone^12,16,23,25,27,29,41,42,50,52,58^. Despite this, no consensus was found regarding the type of question applied. After identifying the questions used in each study, it was possible to group them into seven categories, as described in Table 1.

**Table 1:** Types of questions used in the assessment of motoric cognitive risk syndrome.

Categories	Questions
Memory comparison over time	"Compared to the last two years, how would you rate your memory?" "Is your memory better, the same, or worse than the last interview?" "Is your memory worse than it was ten years ago?"
Perception of memory problems	"Do you feel like you have more memory problems than most people?" "Do you have more difficulty remembering things?"
Impact of memory problems on daily activities	"In recent months, have memory problems affected your daily activities?"
Specific memory difficulties	"Do you forget what you intended to do while on your way to do something?" "Do you have difficulty finding the right words when speaking?"
Memory assessment (rating scale)	"How would you rate your memory currently? Excellent, very good, good, fair, or poor?"
Professional diagnosis	"Has any doctor ever told you that you have a memory problem?"
Assessment of attention and concentration	"How often do you have difficulty keeping your mind on what you're doing?"

Regarding the evaluation of gait slowness, the results were not significantly different. Similarly, it was not possible to identify an assessment protocol or cutoff point to define the value of gait slowness. Additionally, there is no standardized distance for testing, with distances varying between 2.5 and 8.5 meters. In the methodology description, most studies do not clarify whether the authors used instruments that disregard the acceleration and deceleration areas.

## DISCUSSION

This finding suggests a significant reflection on the importance of evaluative protocols, keeping in mind that the term "syndrome" refers to a collection of signs and symptoms occurring simultaneously. Therefore, it is crucial to determine the investigative procedures necessary for identifying a specific clinical condition to recognize clinical signs, monitor the progression of the clinical picture, and propose therapeutic approaches^62^.

Another important observation regarding the results of this scoping review is related to evaluative strategies in subjective cognitive complaints. Historically, the use of single questions to screen cognitive functions began in 2012, when the Ministries of Health in England and Wales suggested this instrument as a strategy to identify individuals at greater risk of developing dementia^63^.

Although this approach is frequently employed in clinical practice, the effectiveness of using a single question as a strategy to identify subjective cognitive complaint remains questionable^64^. In this context, Hendry et al.^65^ conducted a scoping review on using single questions as a screening tool for cognitive function in older individuals; the authors found discrepancies in the formulation of questions, exploring general impressions of memory, confusion, and cognitive function; however, the results suggest that an investigation through a single question can be used in detecting cognitive decline and can be directed either to the patient or to a third party.

On the other hand, recent studies indicate that the use of questions focusing solely on memory may confuse the assessed individuals by neglecting other complaints related to executive functions and language. On this topic, Burmester et al.^66^ conducted a systematic literature review with a meta-analysis to explore how subjective cognitive complaints can indicate impairments in cognitive functioning. They analyzed 53 articles and found that assessments using a single question, like "Do you have problems with your memory?" were common. However, they advised that relying exclusively on a single question could be influenced by confounding variables. They recommended using such an instrument as a screening tool, with further objective testing for individuals reporting cognitive complaints.

Likewise, Sacramento et al.^67^ conducted a cross-sectional quantitative study to investigate memory and self-efficacy changes in older adults. The study revealed that short-term memory, especially working memory, is significantly affected by aging. Furthermore, the authors highlighted the impact of socio-cultural factors on the subjective perception of memory lapses and memory self-efficacy among individuals engaged in active aging. They suggested that beliefs, stigmas, and societal perceptions about older adults can significantly influence these perceptions.

Similarly, Pérez-Blanco et al.^68^ examined whether subjective cognitive complaints reported by informants (caregivers, family) or by individuals themselves are better predictors of MCI progression in older adults. They analyzed seven longitudinal studies involving older individuals without cognitive impairment. Their meta-analysis found that both self-reported and informant-reported complaints are linked to a higher risk of MCI progression. Notably, the association is stronger when reported by a third party, suggesting it provides more reliable information. The authors highlight the need for further research into subjective cognitive complaints to better understand their relationship.

In line with this, Pang et al.^69^ compared the efficacy of three different protocols, all utilizing a single question, in assessing subjective cognitive complaints. They conducted their study within the framework of the "Singapore Epidemiology of Eye Diseases (SEED)" study, recruiting individuals aged 60 years and older. Using the Progressive Forgetfulness Question (PFQ) as a screening tool, they selected 957 eligible participants for further assessment. These individuals underwent evaluations for general cognitive function using the Montreal Cognitive Assessment (MoCA) and a battery of neuropsychological tests. The results showed that, while single-question assessments had lower discriminative rates for detecting dementia compared to MoCA, they suggest the importance of using at least two evaluative instruments (a single question and a screening test) when investigating subjective cognitive complaints.

With respect to the assessment of GS, we observed that, in the selected studies, there was a variation in the chosen protocol about the distance covered and the method applied during the GS assessment. The GS is widely used in clinical practice as it facilitates the investigation of multiple systems such as the cardiopulmonary, neurological, and musculoskeletal systems. Studenski et al.^70^ propose that the clinical application of GS assessment is simple and easy to perform using a stopwatch and a 4-meter track. Individuals start from an initial standing position and are instructed to walk naturally, as if they were walking on a common street, without additional stimulation or instructions.

Also, Wang et al.^71^ investigated the impact of varying distances and the inclusion/exclusion of acceleration and deceleration distances on GS measurements. They divided participants into two groups—young adults and older adults—and measured GS on walkways of 4 and 10 meters, with and without acceleration/deceleration distances. The results showed that GS measurements were comparable across distances only when excluding acceleration/deceleration distances, ensuring stable and consistent measurements.

In accordance, Kirkwood^72^ emphasizes the importance of assessing GS in the multidimensional evaluation of older individuals, as it aids in identifying functional status and health. GS is closely related to functional impairment, cognitive deficit, risk of institutionalization, falls, and mortality. Additionally, the author considers values below 100 cm/s as low GS and suggests that a 4-meter distance is ideal for measuring this variable. It was proposed that the cutoff point of 0.8 m/s for gait speed analysis can be used due to its sensitivity to the health status of older individuals.

Dommershuijsen et al.^73^ conducted a study to establish reference values for GS in older adults living in communities. They analyzed data from 4,656 individuals, finding that GS decreased with age and increased with height, but this effect disappeared above 80 years. They provided specific average GS values by gender and age, with an online platform for accessing these reference values; for instance, at 50 years old, men with average height had a GS of 1.28 m/s, while women had 1.24 m/s. At 90 years old, men's average GS was 0.92 m/s, and women's was 0.91 m/s.

Observing the possible protocols used in gait analysis, Krumpoch et al.^74^ explored the effect of different tests for GS assessment in older adults involving different distances, static versus dynamic tests, and speed instructions; they found significant differences in usual walking speed between static and dynamic tests over 4 meters, with smaller but still significant differences over 8 meters and between distances. Overall, GS was higher without static start/stop, particularly for shorter distances. These findings are crucial for interpreting results in scientific research like systematic reviews and meta-analyses.

### Review strengths and limitations

The strength of this study lies in its status as the first scoping review to investigate evaluation tools used in MRC. However, despite a lack of a well-structured evaluation protocol, not all the studies present the assessment tools with sufficient detail, which poses difficulties for a deeper analysis.

In conclusion, MCR can bring new perspectives to the elderly and their families, as it enables early diagnosis, facilitating access to interventions aimed at enhancing the maintenance of functional capacity, thus offering more functional years and better quality of life. Considering the potential of MCR, more studies focusing on assessment instruments are necessary. Additionally, it is important to develop a diagnostic flowchart and a clinical management flowchart addressing MCR. Finally, these improvements can be useful in disseminating and expanding knowledge on this topic, as well as enabling the implementation of MCR screening in healthcare units.

The results of this review suggest that, although MCR demonstrates efficiency in identifying individuals at higher risk of developing dementia, there is no consensus among authors on which assessment tools should be applied for its identification. Similarly, studies do not specify which instruments are most sensitive for this identification, nor do they present cutoff values to be considered in individual evaluations.
