# Supplementary material for: Evaluation criteria for diagnosing motoric cognitive risk syndrome: a scoping review
Source: Dement Neuropsychol. 2025 May 19;19:e20240208. doi: 10.1590/1980-5764-DN-2024-0208 (PMC12088669; doi:10.1590/1980-5764-DN-2024-0208)
Supplement: Supplementary file 1 [file 1980-5764-DN-19-e20240208-Suppl01.docx]

**SUPPLEMENTARY MATERIAL 1**

**Table S1.** Characteristics of the included studies.

| **Author (year)** | **Country** | **Thematic** | **Population & sample** |
| --- | --- | --- | --- |
| Aguilar-Navarro et al.  (2019)^6^ | Mexico | Prevalence of MCR and progression to cognitive impairment | 726 subjects aged 60 years or older participants from the Mexican Health and Aging Study (MHAS) |
| Ayers et al.  (2019)^9^ | United States | Gait abnormalities in older adults with MCR. | 522 community-dwelling non-demented adults aged 65 and older participants from the Central Control of Mobility in Aging study |
| Bai, et al.  (2022)^15^ | China | MCR and incident disability. | 1754 Community-dwelling adult populations (aged ≥ 60 years) and their spouses, participants from the China Health and Retirement Longitudinal Study (CHARLS) |
|  |  | Prevalence of MCR | 5725 adults, aged over 60 years, participants from China Health and Retirement Longitudinal Study (CHARLS) |
| Beauchet et al.  (2023)^24^ | Canada | Clinical characteristics of MCR and progression to cognitive impairment | 1113 Community-dwelling adult populations Participants from The Quebec Longitudinal Study on Nutrition and Successful Aging” (NuAge) cohort. |
| Beauchet et al.  (2021)^25^ | Canada | Late-life depressive symptomatology in MCR | 1,098 community dwellers aged ≥65 years from the “Nutrition as a determinant of successful aging: The Quebec longitudinal study” (NuAge). |
| Beauchet et al.  (2020)^26^ | Canada | Prevalence of MCR and progression to cognitive impairment | 1113 participants community dwellers aged ≥65 years from the “Nutrition as a determinant of successful aging: The Quebec longitudinal study” (NuAge); |
| Blumen et al.  (2019)^16^ | United States | To identify gray matter (GM) networks associated with MCR. | 89 community-dwelling older adults from the Central Control of Mobility in Aging Study (CCMA) |
|  | United States |  | 89 community-dwelling older adults from the Einstein Aging Study (EAS) |
|  | France |  | 89 community-dwelling older adults from the Gait and Alzheimer and Interactions Study (GAIT) |
| Blumen et al.  (2021)^17^ | United States | To examine cortical thickness, volume, and surface area associated with MCR | 50 older adults from the Central Control of Mobility in Aging Study (CCMA) |
|  | Australia |  | 50 older adults from the Tasmanian Study of Cognition and Gait (TASCOG) |
|  | France |  | 50 older adults from the Gait and Alzheimer's Interactions Tracking study (GAIT) |
|  | Japão |  | 50 older adults from the National Center for Geriatrics e Gerontologia – Estudo das Síndromes Geriátricas (NCGG-SGS) |
| Bommarito, G. et. al (2022)^27^ | Switzerland | To explore the biological substrate of the MCR | 20 older adults, mean age 73.2±6.4 years, referred to the Memory Center of the Geneva University Hospitals for cognitive complaints |
| Bortone et al.  (2022)^18^ | Italy | To stablish the key clinical features of different motoric cognitive risk (MCR) subtypes | 1138 subjects aged 65 years and older |
|  | Italy | To establish the key clinical features of different motoric cognitive risk (MCR) subtypes based on individual quantitativeand measures of cognitive impairment and to compare their predictive power on survival over an 8-year observation time. |  |
|  | Italy | Prevalence of MCR. |  |
| Ceïde et al.  (2022)^19^ | United States | Correlational associations between MCR and inflammatory cytokines (Interleukin 6 (IL-6) and C-Reactive Protein (CRP).  Relationships of apathy and MCR. | 347 community dwelling older adults, age 65 and older, without dementia, participants from the Central Control of Mobility in Aging study (CCMA) |
|  | United States | To investigate the association between apathy and MCR | 542 community-dwelling older adults’ participants from the Central Control of Mobility in Aging study (CCMA). |
| Chen et al.  (2022)^28^ | China | Levels of plasma AD biomarkers (Aβ42 and total tau) and their relationships MCR. | 68 participants 60 years or older |
| Cheng et al.  (2021)^29^ | China | Cognitive performance and physical function in individuals with MCR versus MCI. | 77 participants, free of dementia, were recruited from the neurological outpatient clinic of a medical center in Taiwan |
| Doi et al.  (2022)^30^ | Japão | To examine the association between White matter hyperintensities and MCR | 1227 older adults (mean age: 72.0 ± 6.0 yrs, participants from the National Center for Geriatrics and Gerontology-Study of Geriatric Syndromes (NCGG-SGS) |
| Dreyer-Alster et al.  (2022)^31^ | Israel | To examine the prevalence of the MCR syndrome in multiple sclerosis and its association with disability, disease duration, perceived fatigue, and fear of falling. | 618 female participrants people with multiple sclerosis |
| Felix et al.  (2022)^32^ | United States | MCR incidence and of social support in aging. | 506 community-dwelling older adults |
| George & Verghese, (2020)^33^ | United States | MCR and polypharmacy. | 1119 adults 65 years and older participants from the Health and Retirement Study (HRS), a longitudinal study |
| Groeger et al  (2022)^20^ | Japão | inflammatory biomarkers with MCR. | 1026 participants aged ≥ 60 from the National Center for Geriatrics & Gerontology Study of Geriatric Syndromes (NCGG-SGS) |
|  | United States |  | 351 participants aged ≥ 65 from the Central Control of Mobility in Aging (CCMA) |
|  | Australia |  | 412 Participants aged 60 to 85 from the Tasmanian Study of Cognition and Gait (TASCOG) |
|  | United States |  | 433 Participants aged ≥ 65 years from LonGenity |
|  | United States |  | 879 participants aged ≥ 70 from the Einstein Aging Study (EAS). |
| Jayakody, O. et al. (2022)^34^ | United States | Association between falls and MCR | 522 Participants (age 80.6 ± 5.3 years) free of dementia from The Einstein Ageing Study (EAS) |
| Kravatz et al.(2022)^35^ | United States | Association between olfactory function and MCR | 1119 participants aged 60 years and older from the Rush MAP study |
| Lau et al.  (2019)^36^ | Malasya | MCR risk factors | 1366 older adults (aged 60 years and above) categorized as lowincome from the TUA study |
| Le Floch et al.  (2022)^37^ | France | Association between hypovitaminosis D and MCR | 912 participants from the Gait and Alzheimer Interactions Tracking (GAIT) study |
| Li et al.  (2022)^38^ | China | MCR metabolic mechanisms and signatures. | 6031 Participants from the West China Health and Aging Trend cohort study (WCHAT) |
| Liu et al.  (2021)^39^ | China | To develop and validate a prediction nomogram based on MCR | 1,177 participants from the China Health and Retirement Longitudinal Study (CHARLS) |
| Lord et al.  (2020)^40^ | New Zealand | To exam trajectories of gait and cognition and their association with falls | Life and  Living in Advanced Age Study |
| Lu et al.  (2023)^41^ | China | Relationship between MCR and falls. | 3748 Participants aged ≥ 60 years from the China Health and Retirement Longitudinal Study (CHARLS) |
| Marquez et al.  (2022)^42^ | Colombia | Prevalence of MCR and Risk factors of MCR. | 17577 Participants from the SABE Colombia study (Health, Well-Being, and Aging) |
| Meiner et al.  (2021)^43^ | United States | Risk factors that may be associated with conversion to dementia in MCR | 268 participants from the the Rush Memory and Aging project (MAP) |
|  |  |  | 171 participants from the the Religious Orders Study (ROS) |
| Merchant et al.  (2023)^44^ | China | Association of MCR with body composition, including sarcopenia and systemic inflammation. | 397 pre-frail older adults ≥60 years old from the community |
| Merchant et al.  (2020)^45^ | China | To determine the prevalence and demographics of MCR | 509 Community-dwelling older adults ≥ 60 years |
| Merchant et al.  (2021)^46^ | China | To determine the demographics including physical, functional, and psychosocial  factors in MCR. | 509 Community-dwelling older adults aged ≥60 years |
| Moura, T. G. de, & Pinheiro, H. A. (2021)^12^ | Brazil | Prevalence MCR. | Older adults (age ≥ 60 years) with independent gait and withoutsevere cognitive dysfunctions, who had a record of sociodemographic data, cognitive assessment, functional capacity and gait speed in medical records dated 2017 to 2019. |
| Mullin et al.  (2023)^47^ | Scotland | To assess the prognostic value of MCR for dementia. | 680 community-dwelling participants free from the dementia from Lothian Birth Cohort |
| Mullin, et al.  (2023)^48^ | Scotland | MCR and socioeconomic status, as determined by occupational social class and years of education. | 692 community-dwelling participants free from dementia from the Lothian Birth Cohort |
| Mullin et al.  (2022)^49^ | Scotland | Prevalence and risk factors for MCR | 690 community-dwelling participants free from dementia from the Lothian Birth Cohort |
| Nester et al.  (2020)^50^ | United States | To predict incident MCR | 476 Non-demented MCR-free older adults from the “Central Control of Mobility in Aging study (CCMA) |
| Sathyan et al.  (2019) (a)^51^ | United States | Association between frailty and MCR. | 641 adults, aged 65 and above, from the LonGenity study |
|  |  | To examine polygenic inheritance of MCR | 4915 individuals, age 65 years and above from the Health and Retirement Study |
| Sekhon, Allali & Beauchet,  (2019)^52^ | Canada | Association of ADD and depression with MCR | 29569 participants free from cognitive impairment from the Canadian Longitudinal Study on  Aging (CLSA) |
| Sekhon et al.  (2019)^53^ | France | Compare the characteristics  of older individuals with MCR. | 633 individuals free of dementia, were selected from the cross-sectional Gait and Alzheimer Interactions Tracking” study |
| Shen et al. (2020)^54^ | China | Associations of MCR  and its components with frailty | 429 older adults aged 60 years and older s from the geriatric department of Zhejiang Hospital in China |
| Shim, Kim, & Won, (2020)^55^ | Korea | To examine the association of MCR with cognitive functional domains | 2881 community-dwelling older adults aged 70–84 years from the nationwide Korean Frailty and Aging Cohort Study |
|  |  | Associations between MCR and comprehensive fall-related | 2133 community-dwelling older adults aged 70–84 years, without dementia or any dependence in  activities of daily living from the Korean Frailty and Aging Cohort Study |
| Stephan et al.  (2020)^21^ | United States | To investigate whether five major personality traits are related to the MCR. | 6300 dementia-free older adults aged from 65 to 107 years from the Health and Retirement Study (HRS) |
|  | United States |  | 2083 dementia-free older adults aged from 65 to 107 years from the National Health and Aging Trends Study (NHATS). |
|  | United States |  | 6,785 dementia-free older adults from the Health and Retirement Study (HRS) |
|  | United States |  | 5,665 dementia-free older adults from the Health and Retirement Study (HRS) |
| Udina et al.  (2021)^56^ | United States | To assess differences in dual-task performance between participants with and without MCR | 538 community-dwelling non-demented older adults from the Central Control of Mobility in Aging study |
| Van der Leeuw et al.  (2020)^22^ | United States | To study the association between pain and MCR. | 3244 older adults participating in the Health and Retirement Study |
|  | United States |  | 362 participants in the Central Control of Mobility in Aging Study |
| Verghese et al.  (2019)^23^ | United States | To report clinical predictors of transition to dementia in Motoric Cognitive  Risk Syndrome | 171 n communitydwelling adults age 70 and over from the e Einstein Aging Study (EAS) |
|  | United States |  | 268 participants from the the Rush Memory and Aging project (MAP) |
|  | United States |  | 171 participants from the Religious Orders Study (ROS) |
| White et al.  (2020)^57^ | United States | To characterize the nutritional status, diet quality and individual nutritional components of older adults with MCR. | 25 community-dwelling older adults with  MCR aged 60-89 yrs. |
| Yao et al.  (2023)^58^ | China | To reveal the associations of positive control and aging awareness of SPA with the risk of MCR. | 1137 Chinese community-dwelling older adults. |
| Yaqub et al.  (2022)^59^ | Netherlands | To compare risk factors, neuroimaging characteristics and prognosis of MCR and MCI. | 3025 aged ≥60 years, participants from Rotterdam Study |
| Yuan et al.  (2021)^60^ | China | The prevalence and potential risk factors of MCR | 1592 community dwelling older adults from the Rugao Longevity and Aging Study  (RuLAS). |
| Zhang et al.  (2020)^61^ | China | To explore the prevalence of and factors associated with MCR | 953 Chinese  community-dwelling participants aged ≥ 65 years from the Ningbo Community Study |

Abbreviation: MCR, Motoric Cognitive Risk Syndrome.

**Table S2.** Assessment tools used in the identification of Motoric Cognitive Risk Syndrome.

| **Author (year)** | ***Study Population*** | ***Subjective Cognitive Complaint*** | **Self-reported Question** | **Slow Gate** |
| --- | --- | --- | --- | --- |
| Aguilar-Navarro et al. (2019)⁶ |  | One self-reported question | Compared to the last two years, would you say your memory is? | 4m |
| Ayers et al. (2019)⁹ |  | One self-reported question | Do you feel you have more problems with memory than most? | 5m |
| Bai, et al.  (2022)^15^ | China Health and Retirement Longitudinal Study (CHARLS)^a^ | One self-reported question | How would you rate your memory at the moment? | 4m |
| Bai, et al.  (2022)^15^ | China Health and Retirement Longitudinal Study (CHARLS)^b^ | One self-reported question | How would you rate your memory at the moment? | 4m |
| Beauchet et al. (2023)²^4^ |  | One self-reported question | Do you feel like you have more memory problems than most people? | 4m |
| Beauchet et al. (2021)²^5^ |  | One self-reported question | Do you feel like you have more memory problems than most people? | 4m |
| Beauchet et al. (2020)²^6^ |  | One self-reported question | Do you feel like you have more memory problems than most people? | 4m |
| Blumen et al.  (2019)^16^ | Central Control of Mobility in Aging Study (CCMA)^a^ | Self-reported question and Cognitive Testing | Do you feel you have more problems with memory than most? | 6m |
|  | Einstein Aging Study (EAS)^b^ | Cognitive Testing |  | 6m |
|  | Gait and Alzheimer and Interactions Study (GAIT)^c^ | Self-reported question and Cognitive Testing | Do you feel you have more problems with memory than most? | 6m |
|  | National Center for Geriatrics and Gerontology–Study of Geriatric Syndromes  (NCGG-SGS)^d^ | Self-reported question and Cognitive Testing | Do you feel you have more problems with memory than most? | 6m |
| Blumen et al. (2021)¹⁷ | Central Control of Mobility in Aging Study (CCMA)^a^ | Self-reported question and Cognitive Testing | Do you feel you have more problems with memory than most? | 6m |
|  | Gait and Alzheimer’s Interactions Tracking study (GAIT)^b^ | Self-reported question and Cognitive Testing | Do you feel you have more problems with memory than most? | 6m |
|  | Tasmanian Study of Cognition and Gait (TASCOG)^c^ | Self-reported question and Cognitive Testing | Do you feel you have more problems with memory than most? | 6m |
| Bommarito, G. et al. (2022)²^7^ |  | Cognitive Testing |  | not specified |
| Bortone et al. (2022)¹⁸ | *Salus in Apulia Study^a^* | One self-reported question | Do you feel like you have more memory problems than most people? | 5m |
|  | *Salus in Apulia Study^b^* | One self-reported question | Do you feel like you have more memory problems than most people? | 5m |
|  | *Salus in Apulia Study^c^* | One self-reported question | Do you feel like you have more memory problems than most people? | 5m |
| Ceïde et al. (2022)¹⁹ | Central Control of Mobility in Aging study (CCMA)^a^ | Self-reported question and Cognitive Testing | Do you feel you have more problems with memory than most? | 6m |
|  | Central Control of Mobility in Aging study (CCMA)^b^ | Self-reported question and Cognitive Testing | Do you feel you have more problems with memory than most? | 6m |
| Chen et al. (2022)²^8^ |  | One self-reported question | Do you feel like you have more memory problems than most people? | not specified |
| Cheng et al. (2021)²^9^ |  | Cognitive Testing |  | 10m |
| Doi et al. (2022)^30^ |  | One self-reported question | Do you feel you have more problems with memory than most? | 6.4m |
| Dreyer-Alster et al. (2022)^31^ |  | Cognitive Testing |  | 4.6m |
| Felix et al. (2022)³^2^ |  | Self-reported question and Cognitive Testing | Do you feel like you have more memory problems than most people? | 6m |
| George & Verghese, (2020)³^3^ |  | Two or more self-reported questions | If participants rated their memory as fair or poor | 2,5m |
|  |  |  | if they reported their memory as worse than 2 years ago |  |
| Groeger et al. (2022)²⁰ | Einstein Aging Study (EAS)^a^ | Cognitive Testing |  | 6m |
|  | National Center for Geriatrics & Gerontology Study of Geriatric Syndromes (NCGG-SGS)^b^ | Self-reported question and Cognitive Testing | Do you feel you have more problems with memory than most? | 6m |
|  | Central Control of Mobility in Aging Study (CCMA)^c^ | Self-reported question and Cognitive Testing | Do you feel you have more problems with memory than most? | 6m |
|  | LonGenity Study USA^d^ | Self-reported question and Cognitive Testing | Do you feel you have more problems with memory than most? | 6m |
|  | Tasmanian Study of Cognition and Gait (TASCOG)^e^ | Self-reported question and Cognitive Testing | Do you feel you have more problems with memory than most? | 6m |
| Jayakody, O. et al. (2022)³^4^ |  | Self-reported question and Cognitive Testing | Do you feel you have more problems with memory than most? | 8m |
| Kravatz et al. (2022)³^5^ |  | Two or more self-reported questions | Do you have trouble remembering? And | 2.4m |
|  |  |  | Is your memory worse than 10 years ago? |  |
| Lau et al. (2019)³^6^ |  | Self-reported question and Cognitive Testing | Do you feel you have more problems with memory than most | 6m |
| Le Floch et al. (2022)³^7^ |  | Self-reported question and Cognitive Testing | Do you feel you have more problems with memory than most? | 6m |
| Li et al. (2022)³^8^ |  | Two or more self-reported questions | Do you feel that you have more problems with memory than most? | 4m |
|  |  |  | In the past month, have memory problems affected your daily activities? |  |
| Liu et al. (2021)³^9^ |  | Two or more self-reported questions | How would you rate your memory at the present time? |  |
|  |  |  | Would you say it is excellent, very good, good, fair, or poor? | 2.5m |
| Lord et al. (2020)^40^ |  | One self-reported question | Do you feel you have more problems with your memory than most? | 3m |
| Lu et al. (2023)⁴^1^ |  | Two or more self-reported questions | How would you rate your memory at the present time? | 2.5 |
|  |  |  | Would you say it is excellent, very good, good, fair, or poor? |  |
| Marquez et al. (2022)⁴^2^ |  | Cognitive Testing |  | 3m |
| Meiner et al. (2021)⁴^3^ | Religious Orders Study (ROS)^a^ | Two or more self-reported questions | Ttrouble remembering? | not specified |
|  |  |  | If memory was worse than 10 years ago |  |
|  | The Rush Memory and Aging project (MAP)^b^ | Two or more self-reported questions | Trouble remembering? | not specified |
|  |  |  | If memory was worse than 10 years ago |  |
| Merchant et al. (2023)⁴^4^ |  | One self-reported question | Do you think you have more problems with memory than most? | 4m |
| Merchant et al. (2020)⁴^5^ |  | One self-reported question | Do you think you have more problems with memory than most? | 4m |
| Merchant et al. (2021)⁴^6^ |  | One self-reported question | Do you think you have more problems with memory than most? | 4m |
| Moura, T. G. de, & Pinheiro, H. A. (2021)¹² |  | Cognitive Testing |  | 4m |
| Mullin et al. (2023)⁴^7^ |  | One self-reported question | Do you currently have any problems with your memory? | 6m |
| Mullin, et al. (2023)⁴^8^ |  | One self-reported question | Do you currently have any problems with your memory? | 6m |
| Mullin et al. (2022)⁴^9^ |  | One self-reported question | Do you currently have any problems with your memory? | 6m |
| Nester et al. (2020)^50^ |  | One self-reported question | Do you feel that you have more problems with memory than most? | 8.5 |
| Sathyan et al.  (2019) (a)^51^ | LonGenity study^a^ | Cognitive Testing |  | 6m |
|  | Health and Retirement Study (HRS)^b^ | Two or more self-reported questions | How would you rate your memory at the present time? Would you say it is excellent, very good, good, fair, or poor? | 2.5 |
|  |  |  | Compared with the previous interview, would you say your memory is better now, about the same, or worse than it was then? |  |
| Sekhon, Allali & Beauchet, (2019)⁵^2^ |  | Two or more self-reported questions | How often did you have trouble keeping your mind on what you were doing? | 4m |
|  |  |  | Has a doctor ever told you that you have a memory problem? |  |
| Sekhon et al. (2019)⁵^3^ |  | Cognitive Testing |  | 6m |
| Shen et al. (2020)⁵^4^ |  | One self-reported question | Do you feel you have more problems with memory than most? | 4m |
| Shim, Kim, & Won, (2020)^55^ | The Korean Frailty and Aging Cohort Study (KFACS)^a^ | One self-reported question | Do you feel you have more problems with memory than most? | 7m |
|  | The Korean Frailty and Aging Cohort Study (KFACS)^b^ | One self-reported question | Do you feel you have more problems with memory than most? | 7m |
| Stephan et al.  (2020)^21^ | Health and Retirement Study (HRS)^a^ | Two or more self-reported questions | How would you rate your memory at the present time? Would you say it is excellent, very good, good, fair, or poor? | 2.5 |
|  |  |  | Compared with the previous interview, would you say your memory is better now, about the same, or worse than it was then? |  |
|  | National Health and Aging Trends Study (NHATS)^b^ | Two or more self-reported questions | How would yourate your memory at the present time? |  |
|  |  |  | Would you say it isexcellent, very good, good, fair, or poor? |  |
|  |  |  | would you say your memory is better now, about the same, or worse now than it was then?” | Not specified |
|  |  |  | In the last month, how often did memory problems interfere with your daily activities? |  |
|  | Health and Retirement Study (HRS)^c^ | Two or more self-reported questions | How would you rate your memory at the present time? Would you say it is excellent, very good, good, fair, or poor? | 2.5 |
|  |  |  | Compared with the previous interview, would you say your memory is better now, about the same, or worse than it was then? |  |
|  | Health and Retirement Study (HRS)^d^ | Two or more self-reported questions | How would you rate your memory at the present time? Would you say it is excellent, very good, good, fair, or poor? |  |
|  |  |  | Compared with the previous interview, would you say your memory is better now, about the same, or worse than it was then? | 2.5 |
| Udina et al. (2021)⁵^6^ |  | Self-reported question and Cognitive Testing | Do you feel you have more problems with memory than most? | 8.5m |
| Van der Leeuw et al. (2020)^22^ | Central Control of Mobility in Aging (CCMA)^a^ | Self-reported question and Cognitive Testing | Do you feel you have more problems with memory than most? | 6m |
|  | The Health and Retirement study (HRS)^b^ | Two or more self-reported questions | How would you rate your memory at the present time? Would you say it is excellent, very good, good, fair, or poor? |  |
|  |  |  | Compared with the previous interview, would you say your memory is better now, about the same, or worse than it was then? | 2.5 |
| Verghese et al. (2019)²³ | Einstein Aging Study (EAS)^c^ | Cognitive Testing |  | 6m |
|  | Rush Memory and Aging project (MAP)^d^ | Two or more self-reported questions | Trouble remembering? |  |
|  |  |  | If memory was worse than 10 years ago? | Not specified |
|  | Religious Orders Study (ROS)^e^ | Two or more self-reported questions | Trouble remembering |  |
|  |  |  | If memory was worse than 10 years ago? | Not specified |
| White et al. (2020)⁵^7^ |  | One self-reported question | Do you feel you have more problems with memory than most? | 4m |
| Yao et al. (2023)^58^ |  | One self-reported question | Do you have more difficulty to remember things? | 4m |
| Yaqub et al. (2022)^59^ |  | Two or more self-reported questions | Do you have more difficulty to remember things? |  |
|  |  |  | Are you frequently on your way to do something and then forgetwhat you had intended to do?” | 4m |
|  |  |  | Do you experience difficultyto find the right words when speaking? |  |
| Yuan et al. (2021)^60^ |  | One self-reported question | Do you think you have more memory problems than most people? | 5.5m |
| Zhang et al. (2020)⁶^1^ |  | One self-reported question | Do you think you have more memory problems than most people? | 5m |
